# ‘Do Good, Expect the Worst’: The Indirect Effect of Social Cynicism on Prosocial Behavior via Empathy and Trust

**DOI:** 10.5334/irsp.1009

**Published:** 2025-06-24

**Authors:** Denis Coca, Alin Gavreliuc

**Affiliations:** 1 Department of Psychology, West University of Timisoara, Romania

**Keywords:** trust, empathic concern, perspective-taking, social cynicism, prosociality, structural equation modeling, latent variables

## Abstract

Do cynical individuals still engage in prosocial behaviors when they expect the worst from others? While prior research suggests cynical beliefs reduce empathy and trust—key drivers of prosociality—this pathway remains underexplored. We tested four structural equation models (manifest and latent) using data from 239 Romanian adults. Only the manifest model supported an indirect effect via empathic concern; latent models accounting for measurement error did not. This discrepancy highlights how item-level variance may inflate observed relationships. Theoretical and methodological implications are discussed considering the social axioms model and empathy-trust mechanisms in prosocial behavior.

People across the globe experience a constant and lingering fear of what tomorrow holds. Indeed, feelings of uncertainty reached unprecedented levels during the pandemic, according to the World Uncertainty Index, a measure that tracks global uncertainty by text mining the country reports of the Economist Intelligence Unit across 143 countries (Ahir, Nicholas & Davide 2022). Unfortunately, from the pandemic and the associated cost-of-living crisis to the recent Russia’s invasion of Ukraine and the reignited Israeli-Palestinian conflict, successive shocks have set the tone for a new normal of uncertainty. Against this backdrop of global uncertainty, recent data also shows large, time-persistent, and cross-country heterogeneity in trusting attitudes worldwide. For example, in most European countries people trust each other less than they trust the police (EVS 2022). These results paint the overall picture in rather bleak colors: as the social world around us becomes increasingly uncertain, we may also be less likely to trust and help each other.

Despite this gloomy background, people are still keen on engaging in prosocial behaviors. Approximately three-quarters (72%) of the world’s adult population gave money, time, or helped strangers in 2022 (Charities Aid Foundation 2022). These are just some examples of prosociality, which refer to any voluntary actions between a benefactor and a recipient that are viewed by society as beneficial to the current social order (Dovidio et al. 2017; Hruschka & Henrich 2015). Given the broad spectrum of different behaviors that prosocial responding can entail, some authors suggest that there are four fundamental behavioral aspects of prosociality, namely, helping, sharing, taking care of and feeling empathic with others (Caprara et al. 2005; Caprara et al. 2012; Kanacri et al. 2021).

A large body of research shows that prosociality is linked to many benefits for individuals (Bayram 2016; Caprara, Alessandri & Eisenberg 2012), groups (Baldassarri & Abascal 2020; Eisenberg, Eggum & Di Giunta 2010) and society (Smith 2019). More specifically, a recent meta-analysis revealed that acting prosocially consistently improves individuals’ psychological functioning and eudaimonic well-being, albeit in a modest way (Hui & Hui 2009). Engaging in prosocial acts can also protect individuals’ mental health in times of crisis, with studies showing that they are linked to higher levels of well-being and social support during the COVID-19 pandemic (Haller et al. 2022; Miles et al. 2021) and that helping and caring for others may reduce the deleterious effects of daily stress on emotional well-being (Raposa, Laws & Ansell 2016).

## Links between empathy, trust, and prosociality

Human prosociality is a highly debated concept in psychology, with researchers increasingly interested in understanding which individuals are more likely to engage in prosocial behaviors, as well as the circumstances and recipients of such behavior (Schroeder et al. 2015). Thus, a substantial body of research from within and outside the field of psychology supports the existence of various factors that contribute to prosociality. These factors include individual factors (Caprara, Alessandri & Eisenberg 2012; Kanacri et al. 2021; Pang et al. 2022), situational or contextual factors (e.g., Gavreliuc, Gavreliuc & Semenescu 2021; Gavreliuc, Gavreliuc & Semenescu, 2022; Haller et al. 2022), and cultural factors (Hruschka & Henrich 2015; Sabo & Marinescu 2018; Schroeder et al. 2015). A thorough review of the predictors of prosociality is beyond the scope of the present paper. Instead, we focused only on empathy and trust in the present study, as these constructs were suggested to be reliable predictors of prosociality.

While research on prosociality is undoubtedly substantial and compelling, spanning multiple approaches across various academic fields, there is no clear consensus on the most consistent or strongest predictors of prosociality across cultural contexts. Nevertheless, two of the most mentioned explanations for prosociality and prosocial behavior are the empathy-altruism link and social capital (particularly trust in this study). On the one hand, trust has been suggested as a crucial prerequisite to prosociality, as it was shown to be a core element of interpersonal relationships that can also facilitate interactions between strangers (Jasielska et al. 2021; Zhang 2021). Extensive literature supports the positive link between different measures of trust and various interpersonal outcomes, including cooperation, helping and making donations to others in need (Balliet & Van Lange 2013). On the other hand, it has been suggested that the decision to act prosocially is driven by empathy, defined as the ‘other-oriented emotions elicited by and congruent with perceived welfare of someone in need’ (Batson 2011, p. 11). As such, studies generally hint at the proposition of empathy-induced altruism, with increasing evidence supporting a consistent link between empathy and prosociality (e.g., Batson 2011; Cassels, Chan & Chung, 2010; Choy, Eom & Li, 2021; Eisenberg, Eggum & Di Giunta 2010; Eisenberg, Eggum, & Di Giunta, 2010; Kamas & Preston 2021; Lehmann et al. 2022; Lehmann et al. 2022; Pang et al. 2022; Telle & Pfister 2016).

## Demographic differences in prosociality

In the past, research suggested that women tend to be more prosocial than men, particularly in interpersonal and caregiving contexts. For example, a meta-analysis by Eagly and Crowley (1986) found that women are more likely to engage in nurturing, empathic, and caregiving behaviors, which are often categorized as prosocial. This is thought to be related to socialization processes, where women are encouraged from a young age to be compassionate and community-oriented, aligning with gender norms that emphasize communal roles for women. A more recent study has tried to expand our understanding of gender differences in prosociality, incorporating more nuanced insights. Caprara et al. (2005), who developed the *Adult Prosociality Scale*, provided key evidence that women generally score higher on prosocial traits than men. Their research suggests that women tend to display higher levels of empathy, cooperation, and altruism, which aligns with traditional gender roles emphasizing nurturing and caregiving behaviors. This finding builds on earlier research but refines it by offering a validated scale to measure prosocial tendencies in adults. Therefore, as research suggests there are considerable gender differences in prosociality, we used gender as a covariate and control variable to see if social cynicism could withstand its effect in relationship to prosociality.

Additionally, most studies have focused on the development of prosociality in children and teenagers (Knafo-Noam et al. 2015; Knafo-Noam et al. 2018; Van Der Graaff et al. 2018), and less attention has been given to prosocial response in the later stages of life, notably in adulthood. Some authors argue that prosociality becomes increasingly relevant as individuals age because of the potential effect of alleviating the risks associated with aging (e.g., fewer social interactions) and their contribution to promoting values focused on the welfare of others (Caprara et al. 2005; Caprara, Alessandri & Eisenberg 2012; Kanacri et al. 2021). Consequently, we were only concerned with testing our main hypotheses on adults rather than adolescents or children.

## Social cynicism as a distal antecedent of prosociality

The decision to interact with others in social environments is inherently uncertain owing to the multitude of unknowns (e.g., who to trust, how to express ourselves or whether to help others). It also requires us to generate predictions of others’ thoughts, emotions, and behaviors at any time during our encounters. It is well-known that one motivator of social behavior is the desire to reduce uncertainty (for a review, see FeldmanHall & Shenhav 2019). According to a recent model of social uncertainty developed by FeldmanHall and Shenhav (2019), social stimuli rather than nonsocial situations motivate human social cognition and behavior to a greater extent, as they are more unpredictable, dynamic, and ever-changing (Thornton & Tamir 2017. Substantial evidence from multiple areas of social psychological research also suggests that people typically perceive uncertainty as being aversive and pervasive (e.g., Fiske & Neuberg 1990; Fiske, Lin & Neuberg 1999). FeldmanHall and Shenhav (2019) theorized that individuals are intrinsically motivated to reduce social uncertainty by using three interrelated processes, namely, automatic inferences (i.e., stereotypes or impression formation), controlled inferences (i.e., perspective-taking and empathic concern) and social learning.

It is well known that we, as human beings, are economical in allocating cognitive resources to reduce social uncertainty (Fiske & Taylor, 2013), making great use of mental shortcuts when assessing individuals’ motives and behaviors in social situations (FeldmanHall & Shenhav 2019). One such method is the common use of cognitive anchors as starting points when making inferences about individuals’ traits or social situations. Anchoring is the tendency to make rapid assessments based solely on the first piece of information encountered. It helps us to help make sense of the things around us when faced with uncertainty or time constraints (Furnham & Boo 2011). They can reflect basic premises (or prerequisites) that people endorse or use to guide their behavior across a variety of social situations. As these priors are likely related to social behaviors irrespective of context, individuals, targets, and time periods, we regard social axioms as important anchors in predicting complex social behaviors such as prosocial behavior (Bond et al. 2004; Dincă & Iliescu 2009; Leung et al. 2002). Thus, social axiom represents a mode of implicit cognitive operation, culturally anchored, that may be activated for processing information about situations, events and people (Hui & Hui 2009).

Defined as general beliefs about oneself and the world found in the form of an assertion about the relationship between two entities or concepts (Leung et al. 2002), social axioms are usually assumed to be true through social learning and not objective scrutiny (Bond et al. 2004). From a functionalist point of view, such generalized beliefs are regarded as important for the survival and functioning of humans (Leung et al. 2002; Leung & Bond 2004; Leung & Bond 2009; Leung et al. 2012). Social cynicism, defined as the extent to which people believe in the immoral nature of people and social institutions, may play a particular, yet crucial role in the social domain: it protects individuals from exploitation in an uncertain world. However, holding cynical beliefs does not come without costs. As social cynicism stems from the unfulfillment of high expectations concerning society and social institutions, cynical individuals run the risk of having lower concern for humanity (Leung & Bond 2004), being excluded or ostracized by others (Choy, Eom & Li, 2021), or to suffer losses across other life domains (Hui & Hui 2009). As cynical individuals are less trusting toward others (Kurman 2011) and are less empathetic (Choy, Eom & Li, 2021), we believe that social cynicism may indirectly undermine prosocial responses in adults. Thus, we propose that highly cynical individuals show deficits in generalized trust and empathy toward others, which, in turn, make engaging in prosocial acts less likely to occur.

Trust is a prerequisite to prosocial response in interpersonal relationships, as it may facilitate positive interactions between strangers (Kistler, Thöni & Welzel 2017). While ambiguous and often divergent, definitions of trust widely accept the idea that a ‘party is willing to be vulnerable to the actions of another party on the basis of the expectation of reciprocity, irrespective of the ability to monitor or control the other party’ (Mayer, Davis & Schoorman 1995). Hence, trust can only be demanded in a risky situation. As cynical individuals often lack interpersonal trust and are less likely to engage in trusting behavior (Kurman 2011), it may be reasonable to assume that cynicism represents a lack of trust. However, social cynicism often involves an intense feeling of hostility toward others in addition to a clear reduction in positive expectations, which further differentiates it from generalized trust (Neumann & Zaki 2023). For example, highly cynical individuals tend to distrust and dislike others, are less likely to ask for help when needed, and are more prone to choose coercion, rather than compromise and collaborate during conflicts (see Bond et al. 2004; Hui & Hui 2009). Thus, we expect that trust may act as an explanatory mechanism in the relationship between social cynicism and prosociality.

In addition to showing distrust, cynical individuals often perceive others as morally bankrupt and are perceived as less empathetic (Choy, Eom & Li, 2021; Dincă & Iliescu 2009). Empathy is widely accepted as the range of reactions one has in response to the emotional experiences of others (Davis 1983). The ability to empathize requires one to be attentive to how others feel and to assess their emotional reactions accurately. As prosocial responding often involves an awareness of the need to help someone in distress, empathy is one of the critical antecedents of prosociality (Eisenberg, Eggum & Di Giunta 2010; Kamas & Preston 2021; Pang et al. 2022). Given that cynics tend to dislike the company of others (Hui & Hui 2009), they are also more inclined to treat others disrespectfully (Stavrova, Ehlebracht & Vohs 2020). In line with this reasoning, cynical individuals are less likely to accurately identify and understand others’ emotional needs, which, in turn, can make them less prosocial. Thus, empathy may also act as an important explanatory mechanism in the relationship between social cynicism and prosociality.

To summarize, as both empathy and trust are crucial elements of social functioning, they may stem from generalized social beliefs that people hold to navigate an uncertain social world. Known as social axioms, they serve as both general expectancies about the world and specific principles that motivate goal attainment in life (Leung et al. 2002; Leung & Bond 2004; Leung & Bond 2009). More specifically, we argue that social cynicism, defined as the tendency to negatively portray humanity and humankind, may be a central construct in the nomological network of empathy and trust that is also a distal antecedent to prosociality. However, the link between social cynicism and prosociality has yet to be investigated in such a manner. Therefore, the present study first investigated whether social cynicism acts as a direct or indirect antecedent to prosociality by exploring its links with empathy and trust.

## The current study

Despite the growing interest in the effects of social cynicism on social behaviors, psychological research has largely ignored the potential direct effect of cynicism on prosociality. With few exceptions (Choy, Eom & Li, 2021; Sabo & Marinescu 2018), the social cynicism—prosociality link is largely inconclusive. For example, while social cynicism negatively predicted the amount of money donated to a stranger in a Dictator Game in one study (Sabo & Marinescu 2018), cynicism did not directly predict prosocial behavior in another (Choy, Eom & Li, 2021). Despite its current limitations, existing research hints at the idea that social cynicism may negatively predict prosociality, as cynical individuals are less likely to engage in acts that benefit others due to their gloomy outlook on relationship building.

**Figure 1:**
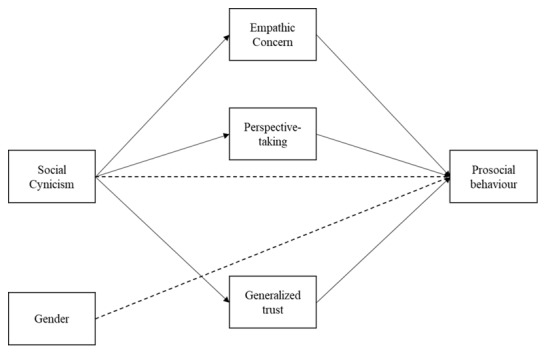
Conceptual model of the present study.

The discussion above hints at the idea that social cynicism may instead undermine prosocial behavior through explanatory mechanisms such as empathy and trust. However, there is no scientific evidence so far to suggest such links. Therefore, the present study aimed to investigate the role of empathy and trust as parallel mediators of the link between social cynicism and prosociality. Conceptually, we theorized that as individuals hold more cynical beliefs, they are also more likely to be less empathetic and less trusting toward others which, in turn, may lower their likelihood of acting prosocially. Finally, we also expected social cynicism to significantly predict prosociality even after controlling gender. Therefore, we proposed the following conceptual model (see Figure 1). Based on the reviewed literature, we expect that:

1. Social cynicism will negatively predict generalized trust, which, in turn, will negatively predict prosocial behavior.
2. Social cynicism will negatively predict both empathic concern and perspective-taking, which, in turn, will negatively predict prosocial behavior.

## Methodology

### Participants

The present study used a cross-sectional design to evaluate the direct and indirect effects of social cynicism on prosociality via empathy and trust. A power analysis was conducted to estimate an adequate sample size by using G*Power 3.1.9.7. (Faul et al. 2009). Using the effect sizes found in previous studies (Choy, Eom & Li, 2021; Sabo & Marinescu 2018), statistical power was estimated for a multiple regression analysis to detect a small to medium effect size (*f*^2^ = .05 with .80 power, α = .05 and a total of four tested predictors). The analysis resulted in *N* = 244 observations needed for such an effect. A total of 266 participants (*M*_age_ = 23.64, *SD_age_* = 5.80) were recruited through snowball sampling via social media. Fourteen responses were discarded because they were underage (*N* = 252). Thus, a total of 252 data points were included in the subsequent statistical analyses. The respondents were predominantly male (31% female), with 1.5% choosing not to specify their gender. In terms of educational level, most of the participants completed high school (51%), while 30% of the participants held at least one university degree.

The participants completed an online questionnaire set on the Google Forms platform. After they were given information on the details of the study, they completed an explicit consent form and the instruments. The completion time was approximately 15 minutes.

### Measures

As previous authors have suggested, Cronbach’s α is less accurate in estimating reliability when the assumption of essential tau equivalence is violated. That is, when not all items measure the same unidimensional factor with the same precision, this is usually the case for multi-item measurement scales (for a review see Hayes & Coutts 2020). Thus, McDonald’s *ω* was used as a related measure that does not assume essential tau equivalence. Reliability coefficients were computed by using the Omega macro for SPSS (Hayes & Coutts 2020). The correlations between the main variables and descriptive statistics for each of the measures presented can be viewed in the **Supplementary Material**.

#### Prosociality

It was measured with the *Adult Prosociality Scale*, whose psychometric properties were replicated and validated on groups of Italian participants (Caprara et al. 2005). See also Kanacri et al. (2021) for further evidence about the validity of the scale. The scale contains 16 statements that are rated on a 5-point Likert scale (1 = not at all/almost never to 5 = always/almost always). The total prosociality score was calculated by summing the individual scores of each item. The McDonald’s *ω* for this scale was .93.

#### Social cynicism

It was measured via the *Social Cynicism* subscale of the 60-item version of the *Social Axioms Scale* (SAS 60; Leung et al. 2012). Participants were asked to rate 18 statements on a 5-point Likert scale from ‘1 = total disagreement’ to ‘5 = total agreement’. The final score for this scale was computed as the average score of all the individual items. The McDonald’s *ω* for this scale was .80.

#### Trust

It was measured with the *Generalized Trust Scale* (GTS; Yamagishi & Yamagishi 1994; Yamagishi, Kikuchi & Kosugi, 1999). GTS presents high reliability and validity in a wide range of social contexts (see Jasielska, Rogoza, Zajenkowska & Russa, 2021 for a more detailed view). Participants rated their responses for six items on a 5-point Likert scale ranging from ‘1 = totally disagree’ to ‘5 = totally agree’. The score was computed as the average score of the individual items. The McDonald’s *ω* for this scale was .77.

#### Empathy

It was measured with the *Interpersonal Reactivity Index* (Davis 1983). Empathy was measured only by empathic concern and perspective-taking subscales from the IRI (13 items). The participants rated their responses on a 5-point Likert scale from ‘1 = does not describe me at all’ to ‘5 = it describes me very well’. The total score for each subscale was computed. The McDonald’s *ω* for the two scales were .76 (Empathic concern) and .80 (Perspective-taking).

Examples of items, as well as the scales used can be consulted in depth in the **Supplementary Material** section of the article. The scales were translated by the author via the back-translation method and were evaluated by a bilingual.

## Results

IBM SPSS v.21 and RStudio (R Core Team 2023) with the *lavaan* package (Rosseel 2012) were used to conduct all analyses, while a structural equation modeling (SEM) approach was used to validate the models and analyze the relationships between the main constructs. The auxiliary analyses (CFA and statistical assumptions) can be found in the **Supplementary Material**.

### Testing the structural models

To assess the robustness of our findings we specified and compared multiple structural models as follows: Firstly, as all main variables showed moderate to high reliability coefficients (≥ .80), **model 1** uses manifest variables. Using composite scores allows for a more parsimonious model while still capturing the intended construct. **Models 2, 3**, and **4** were investigated by using a latent variable approach that accounted for measurement errors and ensured that only well-loading indicators contributed to the constructs. More specifically, in **model 2** all direct and indirect effects were specified, whereas in **model 3** only indirect direct effects were specified between social cynicism and prosociality (full mediation), and finally in **model 4** empathic concern and perspective-taking became the main predictors of prosociality.

#### Model 1

Multivariate normality and model assumptions were verified by investigating variable inflation factors (VIFs) and Cook’s distances. No VIF values greater than 1.5 were observed, indicating that there were no multicollinearity problems while Cook’s distances were equal to or smaller than .015, except for 13 cases which represented multivariate influential points. After a closer inspection, we decided to remove the 13 cases, leaving the final sample size at 239.

**Figure 2:**
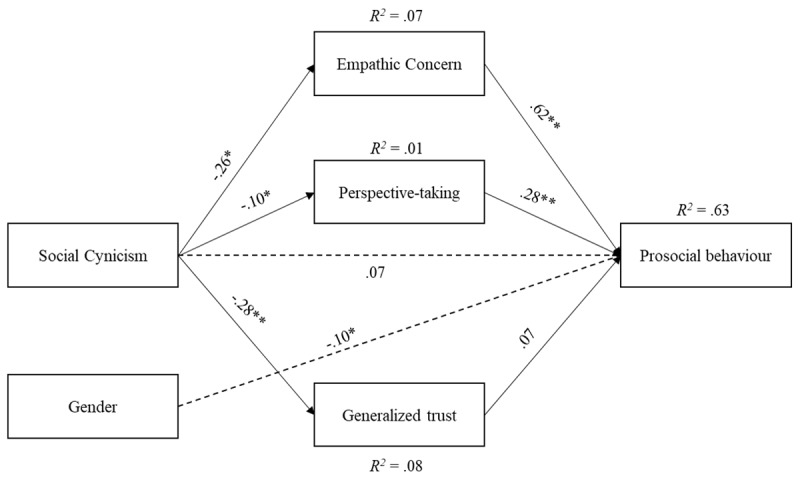
Structural model with standardized path coefficients (Model 1).

Paths were specified according to our hypotheses, while the following variables were covaried: empathic concern (EC) was covaried with perspective-taking (PT). The fit indices showed an overall good fit of the data, *x^2^* = 20.25, *df* = 5, *x^x^/df* = 4.05, CFI = .95, RMSEA = .11, SRMR = .08 (see Figure 2). As expected, *social cynicism* negatively predicted empathic concern (*β* = –.25, *p* < .001) and generalized trust (*β* = –.28, *p* < .001), but not perspective-taking (*β* = –.10, *p* = .093). Empathic concern (*β* = .62, *p* < .001), perspective-taking (*β* = .28, *p* < .001), generalized trust (*β* = .07, *p* = .08), *social* cynicism (*β* = .07, *p* = .085), and gender (*β* = –.10, *p* < .05) directly predicted prosociality. The indirect effect for empathic concern was statistically significant, *β* = –.16 [–.30, –.11). The indirect effect for perspective-taking was not statistically significant, *β* = –.03 [–.07, .01]. Finally, the indirect effect for generalized trust was not statistically significant, *β* = –.02 [–.05, .01]. The model explained approximately 60% of the variance in prosociality (*R^2^* = .63).

#### Model 2

**Figure 3:**
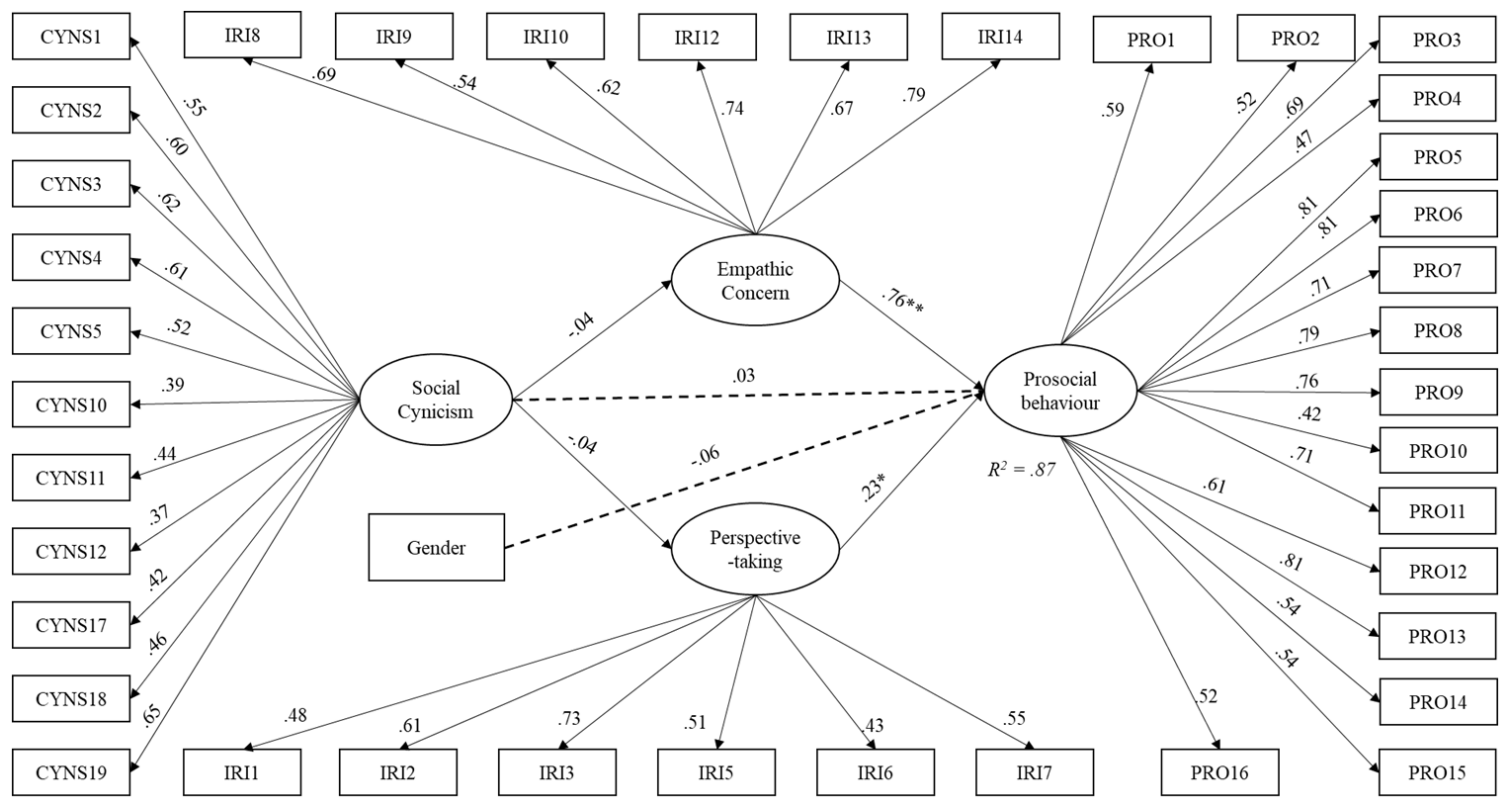
Structural model with standardized path coefficients (Model 2).

Model 2 was specified with latent constructs instead to account for measurement errors due to indicators presenting poor loadings. Specific to model 2, indicators and constructs were covaried as such: CYNS1 with CYN4, IRI6 with IRI7, IRI2 with IRI5, PRO1 with PRO3, PRO2 with PRO14 (due to wording), and Empathic concern with perspective-taking. Generalized trust has been removed. The fit indices revealed an overall acceptable to good fit of the data, *x^x^* = 994.63, *df* = 728, *x^2^/df* = 1.34. CFI = .92, RMSEA = .04, SRMR = .08 (see Figure 3). Social cynicism did not significantly predict neither empathic concern (*β* = –.04, *p* = .606), nor perspective-taking (*β* = –.03, *p* = .658). Both empathic concern (*β* = .76, *p* < .001) and perspective-taking (*β* = .23, *p* < .05) predicted prosociality. However, neither social cynicism (*β* = .03, *p* = .571), nor gender (*β* = –.06, *p* = .10) directly predicted prosociality. Both indirect effects were statistically insignificant, *β* = –.03 [–.12, .07] (for empathic concern), *β* = .01 [–.03, .02] for perspective-taking respectively. The added variables explained approximately 86% of the total variance for prosociality (*R*^2^ = .86).

#### Model 3

**Figure 4:**
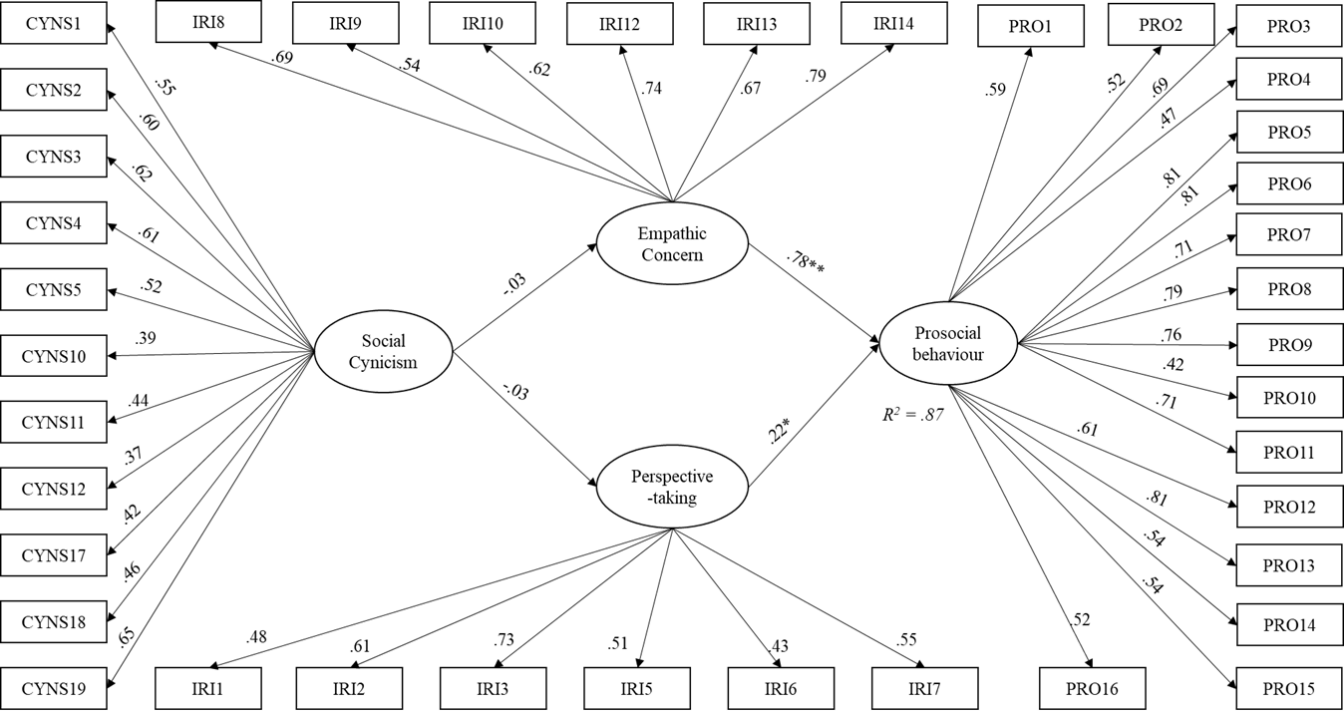
Structural model with standardized path coefficients (Model 3).

Model 3 was identical to Model 2, but the specified direct paths from *gender* and *social cynicism* to *prosociality* have been removed, leaving model 3 to be assessed for full mediation. The same indicators and latent constructs were covaried as in model 2. The fit indices showed an acceptable fit of the data, *x^2^* = 920.93, *df* = 691, *x^2^*/*df* = 1.33, CFI = .93, RMSEA = .04, SRMR = .07 (see Figure 4). Like in model 2, social cynicism did not significantly predict either empathic concern or perspective-taking. Both empathic concern (*β* = .78, *p* < .001) and perspective-taking (*β* = .22, *p* < .05) significantly predicted prosociality. The variables explained approximately 87% of the total variance for prosociality (*R^2^* = .87). No indirect effects were observed for empathic concern, [–.11, .07], nor perspective-taking, [–.03, .02].

#### Model 4

**Figure 5:**
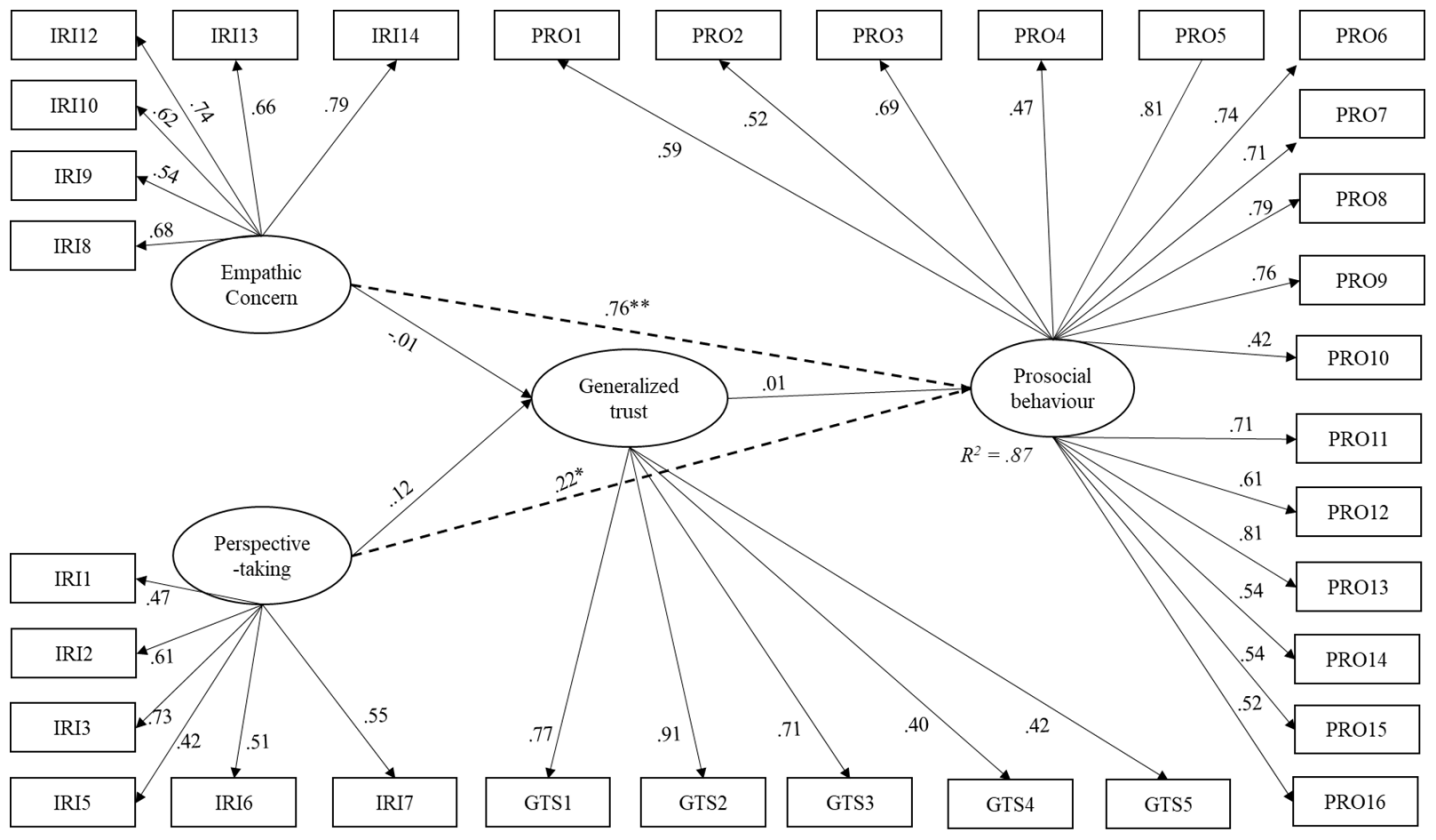
Structural model with standardized path coefficients (Model 4).

Model 4 was investigated by using a latent variable approach where both empathic concern and perspective-taking become predictors of prosociality. Social cynicism has been removed entirely from the model as its path estimates were statistically insignificant. Generalized trust was added to the model as they were shown to increase the model fit for the data. The same indicators and latent constructs were covaried as in model 2. The fit indices show an overall good fit for the data, *x^2^* = 691.83, *df* = 516, *x^2^/df* = 1.34, CFI = .94, RMSEA = .04, SRMR = .07 (see Figure 5). Neither empathic concern (*β* = –.01, *p* = .972) nor perspective-taking (*β* = .12, *p* = .351) predicted generalized trust. Like in all the previous models, both empathic concern (*β* = .76, *p* < .001) and perspective-taking (*β* = .23, *p* < .05) predicted prosociality. Generalized trust did not significantly predict prosocial behavior (*β* = .02, *p* = .693).

## Discussion

The present study aimed to examine the relationships between social cynicism, empathic concern, perspective-taking, and prosociality using a structural equation modeling (SEM) approach. The study’s objective was twofold: first, to test both direct and indirect effects of social cynicism on prosociality and second, to test our findings against other possible theoretical models.

Assessing the comparative model fit across Models 1–4 provides insight into the most suitable representation of the relationships among the study variables. Model 1, which utilized manifest variables, demonstrated a reasonable fit but was more susceptible to measurement error. It initially suggested an indirect effect of social cynicism on prosocial behavior via empathic concern, but this effect disappeared in subsequent models when latent variables were introduced. This attenuation suggests that the relationships observed in Model 1 may have been inflated due to measurement error or specific item variance rather than representing robust theoretical associations. Model 1 also presents a challenge in model fit assessment due to its low degrees of freedom. Research suggests that RMSEA should not be used in models with low df, as it tends to be inflated and misleading (Kenny, Kaniskan & McCoach 2015). The RMSEA for Model 1 suggests poor fit, but this may not be a reliable indicator given the model’s low df. Instead, we focused on CFI and SRMR, both of which indicate a relatively good fit. This suggests that while Model 1 is more susceptible to measurement error, its structural relationships are not necessarily poorly specified.

Model 2 improved upon Model 1 by incorporating latent constructs, thus accounting for measurement errors. The inclusion of latent variables resulted in a notable reduction in the strength of the associations between social cynicism and empathy, raising questions about the robustness of these pathways. Model 3 further constrained the structure by specifying a full mediation model, removing the direct paths from social cynicism and gender to prosocial behavior. While this model provided an acceptable fit, it failed to support indirect pathways from social cynicism to prosociality, suggesting that other unmeasured factors may be at play. Notably, empathic concern and perspective-taking remained significant predictors of prosocial behavior, reinforcing their central role in fostering prosocial actions. Model 4 offered the best overall fit to the data by removing social cynicism altogether and introducing empathic concern and perspective-taking as predictors of prosociality. This model provided the most parsimonious explanation for the data, as indicated by superior fit indices and theoretically coherent pathways. Generalized trust did not significantly absorb the variance in the relationship between either dimension of empathy or prosociality. The removal of social cynicism suggests that its influence on prosocial behavior may be indirect or context-dependent, requiring further investigation. Moreover, the disappearance of the indirect effect of social cynicism on prosociality in latent models underscores the need to carefully consider measurement error when constructing latent variables. Certain items, such as CYNS15 (‘Old people are a heavy burden on society’) and CYNS16 (‘Humility is dishonesty’), appear to be strongly related to empathic concern. This suggests that the observed relationships in Model 1 may have been influenced by item-specific variance rather than reflecting true theoretical associations. This finding further supports past research who suggested the lack of invariance and issues with multi-dimensionality as the main problems for model misfit in the case for social axioms (Bou Malham & Saucier 2014).

As for the overall results, we proposed that cynics’ tendency to discredit others and avoid collaboration with others during conflict resolution (Hui & Hui 2009) would also negatively associate with lowers levels of empathy toward others’ emotional reactions to social phenomena (Choy, Eom & Li, 2021; Dincă & Iliescu 2009; Leung et al. 2002). Our findings provide mixed support for the hypothesized relationships between the constructs. In Model 1, social cynicism was found to negatively predict empathic concern and generalized trust but did not significantly predict perspective-taking. Additionally, both empathic concern and perspective-taking positively predicted prosociality, supporting prior research suggesting that these empathy-related constructs may play a crucial role in fostering prosociality (Kamas & Preston 2021; Lehmann et al. 2022; Pang et al. 2022; Telle & Pfister 2016). Additionally, we found that, in addition to empathy, cynicism was also strongly and negatively related to generalized trust. As trust often requires individuals to be vulnerable to the other party, individuals can risk being exploited by others in social contexts characterized by high levels of uncertainty (FeldmanHall & Shenhav 2019). Thus, social cynicism may act as a protective shell against the threat of exploitation, protecting individuals’ self-interests and emotional needs (Hui & Hui 2009). However, cynical individuals may appear less trusting, and they themselves may trust others less in social contexts that require cooperation and reciprocity to achieve a common goal (Hui & Hui 2009; Kurman 2011). Thus, we also proposed that cynical individuals would be less likely to engage in prosociality via a reduction in generalized trust. This was not the case, as the indirect effect was statistically nonsignificant.

One possible explanation may be attributed to how prosocial behavior was conceptualized in relation to generalized trust. In the present study, individual differences in self-reported prosocial behavior were assessed by using a specific set of voluntary actions relevant for prosocial response in adults, namely, helping, taking care of, assisting and comforting others (Caprara et al. 2005; Caprara et al. 2012; Kanacri et al. 2021). As generalized trust is a general tendency to exhibit positive expectations of others, it may be more strongly associated with measures of reciprocity and trusting behavior than with measures of prosocial behavior. Indeed, some authors have argued that there are conceptual differences between one’s propensity to trust and other-related trust (Zhang 2021). While the former is defined as the extent to which one feels capable of trusting others, the latter is closely related to the feeling that others deserve trust. Thus, it is reasonable to assume that generalized trust may be weakly related to prosocial behaviors that do not incur costs from the benefactor, whereas trustworthiness (other-focused trust) may be more important in interpersonal interactions that do not explicitly imply costs from the benefactor (Zhang 2021). Future research should examine how *trustworthiness* and *propensity to trust*, while closely related, may be driven by different factors or may be related to social interactions differently. For example, Zhang (2021) has suggested that a low propensity to trust might be linked to socially inhibited behavior, while perceiving others of not being worthy of trust might increase the odds of confronting others (Zhang 2021).

The transition to latent variable models (Models 2–4) accounted for measurement errors and allowed for a more refined analysis of construct relationships. Notably, in Models 2–4, social cynicism no longer significantly predicted empathic concern nor perspective-taking, contrasting with findings from Model 1. This suggests that the inclusion of measurement error corrections may attenuate previously observed relationships, raising questions about the robustness of the indirect effects between social cynicism and prosociality. Furthermore, while empathic concern and perspective-taking remained significant predictors of prosociality, neither social cynicism nor gender directly predicted prosociality in Model 2. The absence of indirect effects suggests that social cynicism’s relationship with prosociality may be more complex than initially hypothesized, potentially operating through additional mediators or contextual factors (Choy, Eom & Li, 2021; Sabo & Marinescu 2018).

The present findings contribute to the growing body of literature on the interplay between social cynicism, empathy, and prosociality. The inconsistency of the relationship between social cynicism and empathy across manifest and latent models suggests that future research should carefully consider measurement error when examining these constructs. Measurement errors are a common concern in social axioms research, particularly when using broad, generalized statements that may be interpreted differently across individuals (Bou Malham & Saucier 2014; Leung et al. 2002). Our results highlight that specific items within the social cynicism construct may drive spurious associations and should be examined closely in future research.

Drawing from the literature on the social axioms model (Bond et al. 2004; Hui & Hui 2009; Leung et al. 2002; Leung et al. 2012; Leung & Bond 2009), the present study contributes to the question of whether general social beliefs about the world may negatively associated with lower levels of empathy, trust and prosocial response in adults. While past research has only suggested this idea (Choy, Eom, & Li, 2021; Sabo & Marinescu 2018), this is the first study to investigate both the direct and indirect effects of social cynicism on prosociality. Like other social axioms, social cynicism encompasses axiomatic beliefs about the relationship between two ‘entities’ that are further reinforced by past social experiences, leading to what is commonly known as ‘implicit knowledge’ about how the world works (Leung & Bond 2009). Future research should investigate whether individuals’ cynical beliefs (or other social axioms) are stable over time and if they can lead to decreases in empathy, trust, and prosocial responses in the long run.

Our findings shed some light on the relationship between social cynicism and prosociality. Existing research has shown that highly cynical individuals are less empathetic (Choy, Eom & Li, 2021; Dincă & Iliescu 2009) and have less concern for humanity (Hui & Hui 2009). However, no such study has yet investigated the role of empathy in the relationship between social cynicism and prosociality. Notably, although our structural models also included a reversed order of the predictors, the present findings do not offer conclusive evidence regarding a potential causal order between the main constructs. Moreover, as Model 4 showed the best fit for the data out of all latent models, empathic concern and perspective-taking did not significantly predict social cynicism. Research suggests that less empathetic individuals not only fail to accurately recognize and understand others’ emotional reactions but are also more likely to disrespect others, which, in turn, may elicit more cynical beliefs (Stavrova, Ehlebracht & Vohs 2020), but our study did not find such an effect in the latent models. Researchers should employ longitudinal designs in which cross-lagged interactions between social cynicism and empathy can be tested over time, thus revealing which one precedes the other.

Finally, as the results of this study are based on a specific population living in a context dominated by an inherited pattern of distrust and social cynicism (i.e., Romania), it is crucial to acknowledge how cultural factors might shape the observed relationships between social cynicism, empathy, and prosocial behavior. Given the degree to which many former communist countries have likely inherited a cultural background defined by high levels of interpersonal distrust, low civic engagement and increasing levels of social cynicism (Gavreliuc, Gavreliuc, & Semenescu 2022), there is some evidence to suggest that negative portrayals of human nature may be linked to less prosociality. Social axioms, including social cynicism, are deeply influenced by cultural norms and societal structures (Leung & Bond 2004). For instance, societies with high levels of institutional distrust and historical political instability may foster stronger cynical worldviews, which could, in turn, influence empathic concern and prosocial behavior differently compared to more collectivist and trust-oriented cultures (Bond, Leung, Au, Tong, & Chemonges-Nielson 2004; Bond, Leung, Au, Tong, Lewis, et al. 2004; Dincă & Iliescu 2009; Hui & Hui 2009). Our findings, particularly the attenuation of the indirect effect of social cynicism on prosociality when measurement error was accounted for, may therefore be contingent on the specific cultural background of our sample.

The question of generalizability is further underscored by existing cross-cultural research on prosociality, which suggests that individualistic and collectivistic cultures may differ in how empathy and social attitudes translate into prosocial actions. In collectivist cultures, prosocial behavior is often more group-oriented and context-dependent, meaning that factors like in-group favoritism may moderate the relationship between social cynicism and prosociality (Chopik, O’Brien & Konrath 2017; Smith 2019). In contrast, in individualistic cultures, where personal moral values often drive prosocial acts, social cynicism may be a stronger inhibitor of prosocial tendencies due to its association with skepticism toward others’ intentions (Gavreliuc, Gavreliuc & Semenescu 2021). Future research should thus explore whether the inconsistencies observed in our study hold across different cultural contexts, particularly in societies where systemic distrust is either deeply embedded or relatively absent. By examining these relationships cross-culturally, researchers can better understand whether social cynicism exerts universal effects or whether its role is contextually bound.

In sum, our findings suggest that while social cynicism did not consistently predict empathy in latent models, empathic concern and perspective-taking remain robust predictors of prosociality. Researchers should prioritize construct precision and explore new mediators to better understand how worldview beliefs shape social behavior.

## Data Accessibility Statement

The dataset and syntax, as well as the **Supplementary Material** can be found by accessing the OSF link below (Files tab). https://osf.io/aszhx/?view_only=5718bf2f4d89413d9a0a6ae662a623ca.
